# Nonadditive Entropies and Nonextensive Statistical Mechanics

**DOI:** 10.3390/e27010093

**Published:** 2025-01-20

**Authors:** Ugur Tirnakli

**Affiliations:** Department of Physics, Faculty of Arts and Sciences, Izmir University of Economics, Izmir 35330, Turkey; ugur.tirnakli@ieu.edu.tr

The centennial Boltzmann–Gibbs statistical mechanics [1], which are based on the additive Boltzmann–Gibbs–von Neumann–Shannon entropy [2], have had undeniable success in an extremely large class of physical systems [3]. This theory is generically designed for systems in equilibrium, and is deeply related to chaotic non-linear dynamics [4]. This implies, for classical systems, that the maximal Lyapunov exponent is expected to be positive. However, in many complex systems, where this exponent becomes vanishingly small, the need emerges for non-additive entropies and consistent generalizations of quantities such as the Maxwellian distributions of velocities [5,6], the celebrated Boltzmann–Gibbs weight for energies [7,8], the standard Fokker–Planck equation [9,10,11], and Pesin-like identities [12,13]. As a whole, this amounts to generalized statistical mechanics based on nonadditive entropies [14,15].

One of the possible such generalizations, known in the literature as nonextensive statistical mechanics, was proposed in 1988 by Constantino Tsallis [16] and, since then, has received many applications in the natural, artificial, and social sciences. Nowadays, Professor Constantino Tsallis has an outstanding global impact on physics, astrophysics, geophysics, economics, mathematics, chemistry, and computational sciences, among others (see the bibliography at https://tsallis.cbpf.br/biblio.htm (accessed on 5 November 2024)). In recognition of his extraordinarily creative and productive scientific life and innumerable contributions to the field of statistical physics of complex systems, this Special Issue is dedicated to him on the occasion of his 80th birthday (5 November 2023).

Several manuscripts were selected for publication in this Special Issue, which I will attempt to describe in this article. All of these manuscripts were prepared by researchers who are working all around the world in related areas.

In this Special Issue, we have 21 articles in total, with two of them being review articles and the rest being research articles. Let us now describe these articles briefly.

For the first review article, we have the privilege of having a contribution from C. Tsallis himself [17]. In this article, one can follow the development of the theory over the years, adorned with historical anecdotes. This invaluable contribution can be considered as yet another example in which one can see the progress of new ideas that enable us to understand nature better.

The second review article in this SI is the one where K. Nelson has concentrated on some related open problems [18]. Rephrasing the terminology of the framework based on scale-shape distributions, several interesting remarks and research questions have been discussed.

The contributions of the research articles in this SI starts with [19], which is an application of the Tsallis entropy to an engineering problem. The performance and quantification of uncertainties over the lifetime of a system is critical from engineering point of view. The authors develop a useful criterion for measuring the predictability of the lifetime of a coherent system.

In [20], the authors provide some valuable insights into the interplay between quantum mixing, magic numbers, and thermodynamic properties in many-fermion systems at low temperatures. It is shown that the utilization of Tsallis entropy and an exactly solvable model offer a powerful framework for understanding complex fermionic behavior and its observable consequences.

The contribution to the SI from Liu essentially highlights the crucial role of nonextensive electrons in modifying dust-ion acoustic wave dynamics within collisional dusty plasmas containing negative ions [21]. The obtained results have implications for understanding wave phenomena in various space and laboratory plasma environments where deviations from Maxwellian electron distributions are observed.

In [22], the authors aim to demonstrate the potential for Tsallis entropy analyses of insole pressure data as a quick and accurate tool for identifying vestibular system dysfunction. The proposed detrending algorithm effectively separates balance-related fluctuations from individual walking habits, improving diagnostic accuracy. It is argued that further research with a larger participant pool is needed to validate these findings and refine the diagnostic process.

The next contribution by Pasten et al., in [23], presents a compelling case for the combined use of Tsallis entropy and mutability (dynamical entropy) to analyze seismic sequences and potentially identify pre-earthquake signals. The identified potential indicators offer a promising route for further research in seismic risk assessment and forecasting.

In [24], the authors make a significant contribution to the theory of Dirichlet averages by extending them to the complex domain for matrix-variate cases. This generalization establishes a relationship between Tsallis entropy and Dirichlet averages. It provides a mathematical tool for analyzing and understanding a wide range of phenomena across various disciplines, from special functions and fractional calculus to statistical mechanics and gene expression modeling.

In [25], the reader can find an investigation of the thermodynamic properties of the system created in proton–proton collisions at the Super Proton Synchrotron by analyzing the transverse momentum spectra of identified charged hadrons. Standard Bose–Einstein and Fermi–Dirac distributions are utilized to extract related parameters. These findings are expected to contribute to our understanding of particle production mechanisms and the evolution of the collision system.

In their contributions [26], Shrahili and Kayid offer valuable insights into the past Tsallis entropy of order statistics, providing a framework for understanding and analyzing the uncertainty associated with past events in systems with various structures. Obtained results and derived tools seem to have potential for applications in reliability engineering, lifetime analysis, and broader information theory contexts.

The authors of [27] analyze the transverse momentum spectra of positive pions in high-energy heavy ion collisions using a modified Hagedorn function to extract freeze-out parameters. Their efforts reveal how these parameters depend on both collision centrality and system size. The obtained results are compared to data from the PHENIX and BRAHMS collaborations, and discussed in the context of nonextensive statistical mechanics.

Another interesting contribution by Lenzi et al. [28] demonstrates the significant impact of stochastic resetting on nonlinear diffusion processes. The interplay between these mechanisms leads to non-Gaussian distributions, transient anomalous diffusion, and the emergence of power-law stationary states, which provide valuable insights into systems exhibiting complex diffusion behavior and have potential applications in diverse fields.

In [29], the authors provide a first-principles validation of Fourier’s law in a classical inertial Heisenberg model. The results highlight the relevance of nonextensive statistical mechanics, specifically the stretched *q*-exponential function, in describing the thermal transport properties of complex systems.

Another contribution to this SI investigates Kleiber’s law, which describes the 3/4 power-law relationship between organism mass and metabolic rate [30]. The authors propose a nonlinear dynamical model, grounded in statistical mechanics and renormalization group theory, to explain this law across plant and animal kingdoms. The model uses Tsallis entropy and connects to concepts of rank distributions and conjugate pairs of power-law exponents. The findings offer a unified explanation for Kleiber’s law based on nonlinear dynamics and nonextensive statistical mechanics.

In their article [31], Biro and collaborators explore the mathematical relationships between non-additive entropy formulas such as Tsallis entropy and the Gini index, a measure of inequality. A dynamical model, illustrating the time evolution of the Gini index, is presented.

In [32], Jensen and Tempesta present a group-theoretic approach to classifying entropies, focusing on how the number of system states grows with the number of components. This approach, emphasizing composability and extensivity, leads to a systematic framework encompassing known entropies, such as Boltzmann–Gibbs–Shannon and Tsallis, and introduces new ones. The framework is applied to data analysis, offering improved methods for characterizing complexity in time series data.

Another research article here explores relativistic thermodynamics within the framework of special relativity, examining different viewpoints on how heat and temperature transform under Lorentz boosts [33]. It then investigates the Maxwell–Jüttner distribution, and proposes a connection between the Tsallis distribution, quantum statistics, and the cosmological constant. The study uses de Sitter space-time as a model to achieve this connection, presenting the Tsallis distribution as a deformation of the Maxwell–Jüttner distribution.

In [34], Yoon et al. examine the non-thermal velocity distribution of solar wind electrons. The authors build upon prior research linking this non-thermal distribution to Langmuir turbulence, proposing a model that incorporates whistler-mode turbulence and thermal fluctuations. The model uses a combination of theoretical calculations and particle-in-cell simulations. A key aspect is the consideration of spontaneous thermal fluctuations alongside the background turbulence in shaping the electron distribution. The resulting distribution, determined numerically, exhibits a distinct core and halo electron population, aligning with observational data.

Barauna and collaborators introduce a method for classifying spatiotemporal patterns in complex systems using entropy measures [35]. The authors propose a parameter space based on Shannon permutation entropy and Tsallis spectral permutation entropy to distinguish between various processes. This approach shows promising results in distinguishing various classes of dynamic processes, and paves the way for further research and applications in data-driven science.

Another contribution by Eroglu et al. [36] provides a compelling quantitative analysis of the impact and influence of nonextensive statistical mechanics and the pivotal role of Constantino Tsallis in its development. The study underscores the importance of scientometric methods in understanding the dynamics of scientific knowledge dissemination, and the impact of individual researchers and their collaborations.

In the final article in this SI, the authors provide valuable insights into the statistical properties of Sicilian precipitation data, highlighting the presence of scale-invariant behavior, long-range correlations, and potential climate change impacts [37]. The application of nonextensive statistical mechanics offers a powerful tool for understanding the complexities of evolving precipitation patterns.

We hope that this volume will be of interest not only to physicists, but also to mathematicians and complex systems scientists.
